# Effects of epicatechin, a crosslinking agent, on human dental pulp cells cultured in collagen scaffolds

**DOI:** 10.1590/1678-775720150383

**Published:** 2016

**Authors:** Eun-su Lim, Myung-Jin Lim, Kyung-San Min, Young-Sun Kwon, Yun-Chan Hwang, Mi-Kyung Yu, Chan-Ui Hong, Kwang-Won Lee

**Affiliations:** 1 - Department of Conservative Dentistry, School of Dentistry and Institute of Oral Bioscience, Chonbuk National University, Jeonju, Korea.; 2 - Research Institute of Clinical Medicine of Chonbuk National University, Biomedical Research Institute of Chonbuk National University Hospital, Jeonju, Korea.; 3 - Department of Conservative Dentistry, School of Dentistry, Chonnam National University, Gwangju, Korea.; 4 - Department of Conservative Dentistry, School of Dentistry, Dankook University, Cheonan, Korea.

**Keywords:** Catechin, Collagen, Differentiation, Dental pulp, Scaffold

## Abstract

**Objective:**

The purpose of this study was to investigate the biological effects of epicatechin (ECN), a crosslinking agent, on human dental pulp cells (hDPCs) cultured in collagen scaffolds.

**Material and Method:**

To evaluate the effects of ECN on the proliferation of hDPCs, cell counting was performed using optical and fluorescent microscopy. Measurements of alkaline phosphatase (ALP) activity, alizarin red staining, and real-time polymerase chain reactions were performed to assess odontogenic differentiation. The compressive strength and setting time of collagen scaffolds containing ECN were measured. Differential scanning calorimetry was performed to analyze the thermal behavior of collagen in the presence of ECN.

**Results:**

Epicatechin increased ALP activity, mineralized nodule formation, and the mRNA expression of dentin sialophosphoprotein (DSPP), a specific odontogenic-related marker. Furthermore, ECN upregulated the expression of DSPP in hDPCs cultured in collagen scaffolds. Epicatechin activated the extracellular signal-regulated kinase (ERK) and the treatment with an ERK inhibitor (U0126) blocked the expression of DSPP. The compressive strength was increased and the setting time was shortened in a dose-dependent manner. The number of cells cultured in the ECN-treated collagen scaffolds was significantly increased compared to the cells in the untreated control group.

**Conclusions:**

Our results revealed that ECN promoted the proliferation and differentiation of hDPCs. Furthermore, the differentiation was regulated by the ERK signaling pathway. Changes in mechanical properties are related to cell fate, including proliferation and differentiation. Therefore, our study suggests the ECN treatment might be desirable for dentin-pulp complex regeneration.

## INTRODUCTION

Apexification has been used to treat pulp pathosis in immature teeth with open apices. The disadvantage of apexification is that it neither allows the root wall to thicken nor continuously develops the immature root^19^. To overcome these disadvantages of apexification, regenerative endodontic therapy was introduced^10^. In this approach, a blood clot serves as a scaffold for the growth and differentiation of stem cells. However, there have been many controversies over regenerative endodontic therapy regarding the nature of the regenerated tissue and its predictability^22^. Meanwhile, as biotechnology has developed there have been many attempts to regenerate pulp-dentin complexes using tissue engineering concepts.

Tissue engineering treatments involve stem cells, scaffolds, and suitable growth factors. Among these, three-dimensional scaffolds offer an adhesive substrate for cultured cells and a physical support to form organs. Since collagen is an element present in all tissues and non-toxic materials, it is a common and useful scaffold. In particular, the dentin organic matrix is described as a macromolecular complex of which approximately 90% is composed of collagen^6^. In addition, collagen is available as a hydrogel scaffold, which can be applied in a small and narrow prepared root canal space. This feature of collagen hydrogels suggests the appropriateness for its use in regenerative endodontic therapy^18^. In this respect, a collagen hydrogel scaffold is considered useful for the regeneration of the pulp-dentin complex. Although collagen is suitable for regenerative therapy, it has disadvantages such as weak physical properties and rapid biodegradation. Therefore, various crosslinking agents have been used to overcome these shortcomings; one of them is glutaraldehyde. Glutaraldehyde is cheaper and has a quicker reaction time than collagen but causes severe toxicity at the site of application because of the release of unreacted aldehydes^9^. Thus, less toxic crosslinking agents must be investigated.

**Figure 1:**
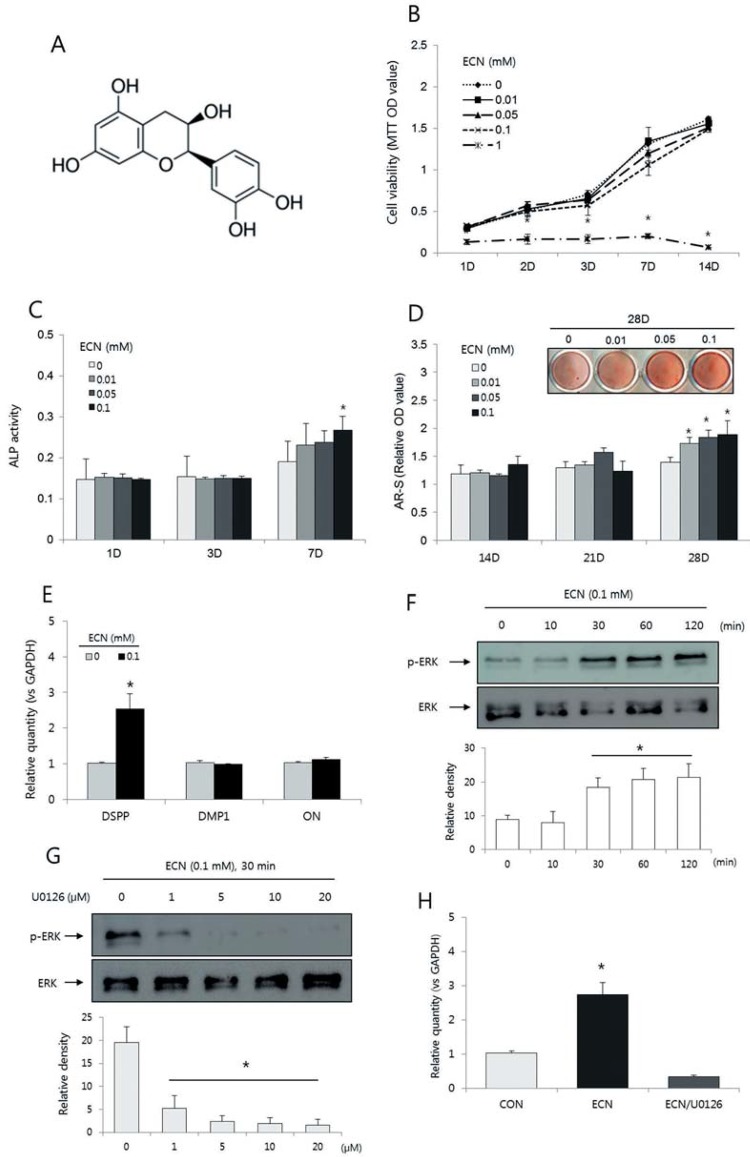
Effects of epicatechin (ECN) on odontogenic differentiation of human dental pulp cells (hDPCs). (A) Chemical structure of ECN. Effects of ECN on cell viability measured by the MTT assay (B), the ALP activity (C), and the formation of calcification nodules (D) in hDPCs. *A significant difference compared with the control was determined at p<0.05. (E) Effects of ECN on the expression of DSPP, DMP1, and ON mRNA in hDPCs cultured for 7 days. (F) p-ERK expression increased within 30 minutes. (G) p-ERK expression was effectively blocked by 5 μM of U0126. (H) The expression level of DSPP mRNA induced by ECN (0.1 mM) was blocked by U0126 (5 μM) after 7 days. The relative levels of gene expression were normalized against GAPDH, and the control level was set as 1.0. *Significant difference compared with the control (p<0.05)

Epicatechin (ECN) is a naturally produced flavanol found in cacao and green tea, and has been orally ingested safely by humans for many centuries (Figure 1A) ^17^. Epicatechin and its derivatives are known to have great potential for anti-inflammatory^7^ and anti-cancer effects^4^ ^,^ ^8^. Furthermore, they are also used as crosslinking agents^3^ ^,^ ^15^. However, there have been no studies regarding the effect of ECN on human dental pulp cells (hDPCs) or on the cells cultured in a collagen hydrogel scaffold. Therefore, the aim of this study was to investigate the effects of ECN on the proliferation and differentiation of hDPCs and the physical properties of collagen, as well as its suitability as a scaffold for regenerative endodontic therapy. We also briefly examined the role of extracellular signal-regulated kinase (ERK) as a mediator of ECN-induced differentiation in hDPCs.

## MATERIAL AND METHODS

### Primary culture of hDPCs

Freshly extracted human third molars were obtained from the Chonbuk National University Dental Hospital in Jeonju, Korea. The teeth were sectioned, and dental pulp tissue was removed aseptically. The tissue was minced into small fragments, which were then cultured in minimal essential medium-α (HyClone Laboratories, Logan, UT, USA) containing 10% of fetal bovine serum (Invitrogen, Carlsbad, CA, USA) along with 100 U/mL of penicillin and of 100 U/mL streptomycin (Invitrogen). Cultures were incubated at 37°C in a humidified atmosphere of 5% CO_2_ and 95% air. Cell cultures between the third and fifth passages were used in this study. All experimental procedures were approved by the Institutional Review Board.

### Cell viability tests

The hDPCs were seeded in 24-well culture plates at a density of 2×10^4^ cells *per* well and incubated in growth medium for 24 h. Then, the cells were exposed to various concentrations (0, 0.01, 0.05, 0.1, and 1 mM) of ECN (Sigma-Aldrich, St. Louis, MO, USA) for up to 14 days. Cell viability was evaluated using the 3-(4,5-dimethylthiazol-2-yl)-2,5-diphenyltetrazolium bromide (MTT) assay. Briefly, 200 mL of MTT solution (0.5 mg/mL in PBS) was added to each well and the wells were incubated for 2 h. Subsequently, 200 mL of dimethyl sulfoxide (DMSO; Amresco, Solon, OH, USA) was added to each well. The plates were shaken until the crystals had dissolved and the solution in each well was transferred to a 96-well tissue culture plate. Reduced MTT was then measured spectrophotometrically at 540 nm in a microplate reader (SPECTROstar Nano, BMG Labtech, Ortenberg, Germany).

### Alkaline phosphatase (ALP) activity

The hDPCs were seeded in six-well culture plates at a density of 2×10^4^ cells per well and were incubated for 24 h for the initial attachment. After the cells were incubated for one, three, or seven days in the presence of various concentrations of ECN (0, 0.01, 0.05 or 0.1 mM), they were scraped into cold PBS (HyClone Laboratories). The ALP activity in the supernatant was determined according to the SensoLyte pNPP ALP Assay Kit (AnaSpec Inc, Fremont, CA, USA) using a modified method established in a previous study^16^. The absorbance was measured at 405 nm using a microplate reader (SPECTROstar Nano).

### Alizarin red S staining

The hDPCs were distributed in a 24-well culture plate at a density of 2×10^4^ and cultured for 24 h. Then, the cells were treated with different concentrations of ECN for the duration of the experiment. After the hDPCs were cultured for 14, 21, and 28 days, the cells were washed with PBS and reacted with 40 mmol/L of alizarin red S solution (Sigma-Aldrich) adjusted to a pH of 4.2 with ammonium hydroxide. After being washed with distilled water, the samples were reacted with 10% cetylpyridinium chloride solution (Sigma-Aldrich) at room temperature for 15 min to dissolve the stain. The contents from each well were transferred to a 96-well plate and were measured spectrophotometrically in a microplate reader (SPECTROstar Nano).

### Gene expression analysis of real-time polymerase chain reaction (PCR)

After exposure to ECN for 7 days, the hDPCs cultured in collagen were lysed directly in the plates using 1.0 mL Trizol reagent (Invitrogen). After chloroform extraction, the total RNA was recovered from the aqueous phase and precipitated with isopropanol and RNAase-free distilled water. Then, reverse transcription of RNA was performed using a Superscript First-Strand Synthesis kit (Invitrogen). Thereafter, the reverse transcriptase-generated first-strand DNA was amplified.

Reactions for real-time PCR were prepared as follows: 12.5 μl of QuantiTect SYBR Green PCR master mix (Qiagen, Hilden, NRW, Germany), 2.5 μl of each primer, and 8 μl of distilled water were used *per* 25-μl reaction volume. Two microliters of template were added to each of the tubes. Samples were always measured in triplicate, while standard curve samples were only single measured as triplicate dilution series were prepared. Primers used for real-time PCR were purchased from Qiagen: dentin sialophosphoprotein (DSPP; catalog #QT00061887), dentin matrix protein 1 (DMP1; catalog #QT00022078), osteonectin (ON; catalog #QT00018620), and glyceraldehyde 3-phosphate dehydrogenase (GAPDH; catalog #QT00079247). Real-time PCR was performed in a Rotor-Gene Q real-time PCR machine (Qiagen) in the 72-well rotor. The thermocycling program for real-time PCR was as follows: 5 min at 95°C for the initial denaturation, followed by 40 cycles of 10 s at 95°C, 10 s at 60°C, and 20 s at 72°C. Finally, a melting curve ranging from 75 to 95°C was performed with increments of 1°C.

### Western blotting

The hDPCs were distributed in 60 mm plates with consistent density. Cells were then incubated in the presence of ECN (0.1 mM) for 0,10, 30, 60, or 120 min. Cells were washed using 1×PBS (Hyclone Laboratories) and were scraped using the cell scraper. A protein lysis buffer was used for 10 min on ice to solubilize the cells. The resolved solutions were centrifuged at 13,000 rpm for 10 min, and the supernatants were transferred to other tubes. Protein concentrations were measured using the Bradford reagent (Bio-Rad Laboratories, Hercules, CA, USA). Analyzed proteins were separated with 9% sodium dodecyl sulfate-polyacrylamide gel electrophoresis and transferred to nitrocellulose membranes (Protran; Whatman, Dassel, Lower Saxony, NI, Germany). Membranes were blocked with 5% skim milk in Tris-buffered saline and Tween 20 for 30 min at room temperature. After being washed three times, the membranes were incubated overnight at 4°C with primary antibodies against p-ERK (Cell Signaling Technology, Danvers, MA, USA) or ERK (Cell Signaling Technology), followed by incubation with horseradish peroxidase-conjugated secondary antibodies. Separated proteins were detected using the ECL Western Blotting Luminol agent (Santa Cruz Biotechnology, Santa Cruz, CA, USA).

### Effect of ERK inhibition on expression of odontoblastic/osteoblastic markers

The hDPCs were cultured in 60 mm plates for 7 days with or without 5 μM U0126 (Promega, Madison, WI, USA), an inhibitor of p-ERK. Cells were pre-incubated with U0126 for 1 h before treatment with ECN. The inhibitor was dissolved in DMSO, and control cells were pre-incubated with equivalent amounts of DMSO alone.

### Preparation of collagen scaffolds

The collagen scaffold was prepared by using the method described previously^13^. Briefly, a purified bovine collagen solution (PurCol; Advanced BioMatrix Inc., Tucson, AZ, USA) and 10×PBS were mixed in an 8:1 ratio and adjusted to a pH of 7.4 with 1 mL of sodium hydroxide (1 M) (Bio Basic Canada Inc., Markham, ON, Canada). The mixture was incubated at 37°C for 2 h further.

### Cell number measurement

Six-well culture plates were coated with 1.5 mL of collagen solution with or without ECN (n=10). After collagen gelation, the hDPCs (1×10^5^) were seeded onto the specimens and cultured for 3 days. The cells cultured in half of the plates were stained with 4’,6-diamidino-2-phenylindole (DAPI) (Invitrogen). Then, the cell numbers were determined under an optical (Leica, Solms, Hessen, HE, Germany) (n=5) or fluorescent microscope (Carl Zeiss, Jena, Thuringia, Germany) (n=5) by two calibrated examiners who did not have any information regarding the experiment. Briefly, a rectangle was inscribed in a round six-well plate and divided into nine equal areas. Then, three images were acquired from each area, and the number of cells [mean ± standard deviation (SD)] in each area was counted by the examiners.

### Setting time

The setting times of the collagen scaffolds were measured using the tilting method described previously^18^. Purified bovine collagen solution was mixed with 10×PBS at a ratio of 8:1 (volume). The pH was adjusted to 7.4 by adding 1 mL of sodium hydroxide (1 M) (Bio Basic Canada Inc., Markham, ON, Canada). Then, the mixture was treated with 0 or 1 μM of ECN and inserted into an Eppendorf tube. We considered the hydrogel collagen to be set if it did not flow when the tube was tilted 45°C. The samples (n=5) were tested just before their anticipated setting times and at 30 s intervals.

### Compressive strength

The compressive strength was evaluated using the method described previously^13^. Round polymerized collagen samples (1 mm×2.5 mm) treated with ECN (0, 0.01, 0.05, or 0.1 mM) were prepared (n=5). Then, their compressive strengths were measured using a universal testing machine (TMS-Pro; Food Technology Corp., Sterling, VA, USA) with a 0.5-N load cell moving at a crosshead speed of 1 mm/min.

### Differential scanning calorimetry (DSC)

A differential scanning calorimeter (Q20; TA Instruments, New Castle, DE, USA) was used to determine the thermal behavior of the crosslinked collagen scaffolds. The collagen scaffolds were examined under wet conditions. The heating was carried out at 10°C/min under nitrogen air in a temperature range from 30 to 330°C. The denaturation temperature was determined as the peak value of the corresponding endothermic phenomena and the denaturation enthalpy was calculated as the area of the peak regarding the weight of collagen.

### Statistical analysis

Statistical analysis was performed using the Student’s t-test or the one-way analysis of variance (ANOVA), followed by a multiple-comparison test (Tukey’s test). A *p*-value smaller than 0.05 was considered statistically significant.

## RESULTS

### Cell viability test

We evaluated the effects of ECN on cell viability using an MTT assay. As shown in Figure 1B, hDPCs treated with 0.01, 0.05, or 0.1 mM of ECN did not show any statistically significant difference in viability compared to the cells in the control group (p>0.05). However, the cells treated with 1 mM of ECN showed significantly lower viability (p<0.05). Based on this result, we selected the concentration of 0.1 mM for use in this study.

### Alkaline phosphatase (ALP) activity

Alkaline phosphatase activity was measured as an early marker of odontogenic differentiation of hDPCs. There were no significant differences between the ECN-treated cells and control cells through day 3 (p>0.05). However, on day 7, the ALP activity increased in the cells treated with 0.1 mM of ECN (p<0.05) (Figure 1C).

### Alizarin red S staining

We evaluated the mineralization of the extracellular matrix using alizarin red S staining. The formation of mineralized nodules in the ECN-treated cells was significantly higher than that in the control group cells on day 28 (p<0.05; Figure 1D).

### Expression of odontogenic markers in hDPCs *per se*

Dentin sialophosphoprotein, DMP1, and ON served as odontogenic markers. As shown in Figure 1E, the expression of DSPP mRNA increased in the presence of 0.1 mM of ECN on day 7.

### Effect of the ERK inhibitor on ECN-induced odontogenic marker expression

Epicatechin (0.1 mM) caused the accumulation of p-ERK in hDPCs within 30 min of treatment (Figure 1F). As shown in Figure 1G, 5 μM of U0126 effectively blocked the expression of p-ERK after 30 min. To address whether the ERK pathway influences odontogenic differentiation, real-time PCR was performed on hDPCs treated with 0.1 mM ECN in medium with or without 5 μM U0126 for 7 days. The results showed the expression of DSPP decreased significantly with U0126 treatment in ECN-treated hDPCs (Figure 1H).

### Cell number measurement

**Figure 2:**
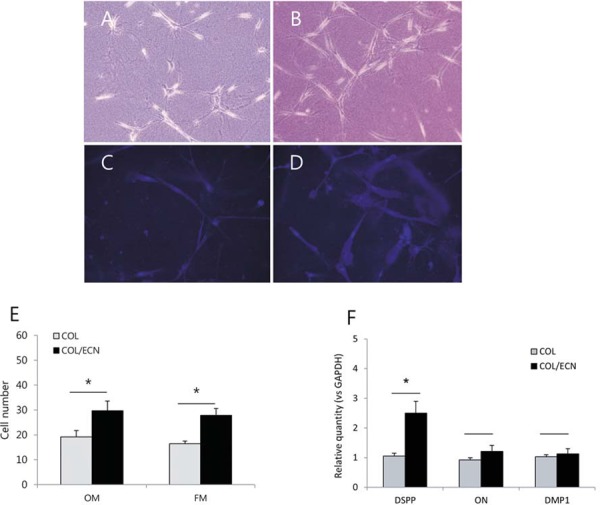
Proliferation analysis of human dental pulp cells (hDPCs) cultured in collagen scaffolds. Optical microscopic views of hDPCs cultured in untreated (A) and epicatechin (ECN)-treated collagen matrices (B) (×200). Representative fluorescence microscopy images (×200) showing hDPCs (stained with DAPI) growing in the collagen scaffold after 3 days of culture in the absence (C) or presence (D) of epicatechin. (E) Effects of ECN-induced crosslinking on proliferation of hDPCs cultured in collagen; OM: optical microscope, FM: fluorescence microscope. (F) The effects of ECN on the expression of DSPP, DMP1, and ON mRNAs in hDPCs cultured in collagen scaffold for 7 days. The relative gene expression levels were normalized against GAPDH and the control was set as 1.0.*Significant difference compared with control (p<0.05)

Cell numbers were measured under optical (Figures 2A and B) and fluorescence microscopes (Figures 2C and D). The number of cells in the ECN-treated crosslinked group was significantly higher than that of the untreated control group (p<0.05; Figure 2E).

### Expression of odontogenic markers in hDPCs cultured in collagen scaffolds

We assessed whether the collagen crosslinking using ECN promoted the expression of odontogenic markers in hDPCs cultured in collagen scaffolds. As shown in Figure 2F, the expression of DSPP mRNA was higher in the cells cultured in the ECN-treated collagen compared to the cells in the control group (p<0.05).

### Setting time and compressive strength

**Figure 3:**
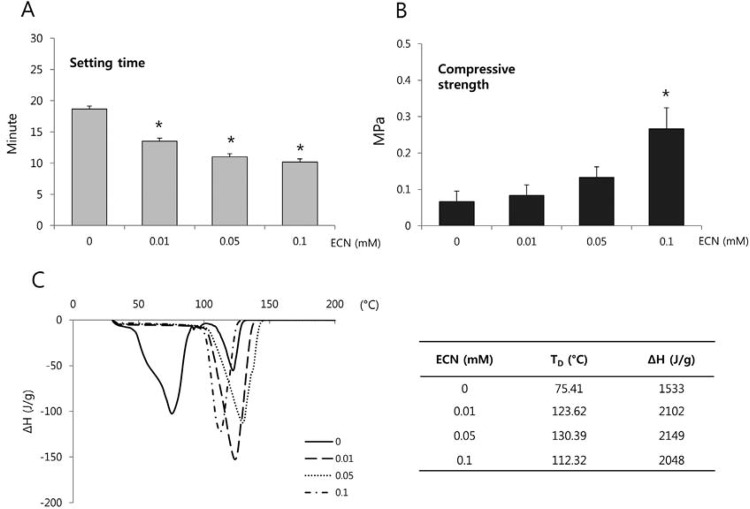
Setting time (A) and compressive strength (B) of collagen in the presence of various concentrations of epicatechin (ECN). (C) Differential scanning calorimetric curves of uncrosslinked and crosslinked collagen scaffolds. *Significant difference compared with control (p<0.05)

The setting times of the ECN-treated collagens were significantly lower than those of the untreated controls (p<0.05; Figure 3A). The compressive strength of the cross-linked collagen increased in a dose-dependent manner. However, only the 0.1 mM ECN group exhibited a significant difference in compressive strength compared to the untreated control group (p<0.05; Figure 3B).

### Differential scanning calorimetry

Differential scanning calorimetry studies were undertaken to evaluate the thermal behavior of collagen scaffolds, and the thermograms are displayed in Figure 3C. The thermogram of the uncrosslinked collagen exhibited an endothermic sharp peak at 75.41°C due to its denaturation, whereas the collagen crosslinked with 0.1 μM ECN exhibited a peak at 112.32°C.

## DISCUSSION

Crosslinking of collagen scaffolds is considered a favorable technique because it enhances many beneficial properties of the scaffold and may provide some advantages concerning cell growth and fate. Recently, several naturopathic medicines are gaining attention for collagen crosslinking because of their low cytotoxicity compared to glutaraldehyde^2^ ^,^ ^3^ ^,^ ^5^ ^,^ ^11^ ^,^ ^13^. Among these, ECN and its derivatives have been reported for having various pharmacologic functions including anti-inflammatory and anti-cancer effects. However, there was no previous information regarding the odontogenic effect of ECN in pulp cells for dentin-pulp complex engineering. Therefore, we investigated whether ECN, a collagen cross-linking agent, promoted odontogenic differentiation of hDPCs.

We examined the effects of ECN on the odontogenic differentiation of hDPCs in this study. Treatment with 0.1 mM of ECN increased ALP activity, calcium nodule formation, and expression of DSPP mRNA, which is considered a specific biochemical marker of functional odontoblasts (Figure 1C-E).^1^ Zeng, et al.^23^ (2014) demonstrated the ECN promoted osteoblast differentiation and might have beneficial effects on bone loss prevention. Wei, et al.^21^ (2011) also showed the catechin stimulates osteogenesis in mesenchymal stem cells. Furthermore, Kwon, et al.^13^ (2015) recently introduced genipin, another collagen crosslinking agent, which has stimulatory effects on odontogenic differentiation, and suggested that cross-linking of collagen may be advantageous for pulp-denin complex regeneration. In this respect, the application of ECN might be beneficial for the odontogenic differentiation of hDPCs incorporated in collagen scaffolds for use in regenerative therapy.

Then, we investigated the mechanism of ECN for the differentiation of hDPCs. Since the ERK pathway has been reported as an important mechanism for odontogenic differentiation in hDPCs^12^ ^-^ ^14^, we postulated that ERK might play a significant role in ECN-induced odontogenic differentiation. As shown in Figure 1F, 0.1 mM of ECN increased the phosphorylation of ERK within 30 min. Furthermore, the blocking of the ERK signaling by U0126, a specific antagonist of ERK, inhibited the expression of DSPP, the odontology-related gene that was upregulated by treatment with ECN (Figure 1H). Based on these experimental results, it is reasonable to assume the ERK is a regulator of the ECN-induced odontogenic differentiation of hDPCs.

Next, we evaluated the effect of ECN treatment on the proliferation and differentiation of hDPCs cultured in collagen hydrogel scaffolds. As shown in Figure 2A-E, the number of cells in the crosslinked group was statistically higher than that of the uncrosslinked control group. Similar to the aforementioned real-time PCR results, the expression of DSPP was increased in the cells cultured in ECN-treated collagen compared with the cells in untreated collagen (Figure 2F). In this study, two different types of microscopes were used to accurately count the cell numbers. Actually, it is difficult to count cells cultured in collagen scaffolds using optical microscopes because of the overlying collagen. To solve this problem, these cells were stained with DAPI to increase their contrast with the scaffolds. We double-checked the effect of ECN on the proliferation of hDPCs using two different microscopes, and the results were similar. Although the effect of ECN on the differentiation of hDPCs was predictable, the reason for the increasing in cell growth is not clear.

We hypothesized that the quicker setting time and enhanced mechanical properties of the crosslinked collagen contributed to the increased cell proliferation. To verify this hypothesis, setting times and compressive strengths were assessed as mechanical properties. We found the setting time was significantly shortened by treating the collagen with ECN (Figure 3A). The gel phase of hydrogel provides a more suitable environment for cells to attach and grow compared to the sol phase. The faster conversion of collagen hydrogel into the gel phase might provide more opportunities for the cells to attach to the substrate. Furthermore, the compressive strengths of ECN-treated collagen scaffolds were higher than those of the untreated collagen, increasing in a dose-dependent manner (Figure 3B). Mechanical properties are important for maintaining a three-dimensional architecture for cell attachment and migration^20^. Zoldan, et al.^24^ (2011) suggested that mechanical stimuli play an important role in stem cell differentiation. As the addition of more crosslinking agents improves the mechanical properties of collagen scaffolds, this might contribute to cell proliferation and differentiation^13^. According to the previous studies and the current results, we suggest that a quicker setting time and increased compressive strength of collagen scaffolds, which were achieved by ECN-induced crosslinking, are beneficial to the proliferation and differentiation of hDPCs.

Lastly, DSC measurement was performed to investigate the thermal behavior of collagen scaffolds. The denaturation point is a physical parameter used to identify the nature of a substance. As shown in DSC results (Figure 3C), the enthalpy of samples denaturation (∆H) was determined by the peak in the DSC curve. The higher melting peak in the crosslinked collagen samples indicates the samples consumed more energy for denaturation than the uncrosslinked collagen samples. Therefore, the application of ECN into the scaffolds stabilizes the substrate based on the molecular interaction between the collagen and ECN, and this stabilization increases cell proliferation along with the aforementioned mechanisms. In addition, this property might be beneficial for preventing the degradation of collagen scaffolds used in future *in vivo* studies.

## CONCLUSIONS

Collectively, our results suggest the crosslinking of collagen with ECN positively affects the attachment, proliferation, and differentiation of hDPCs cultured in collagen scaffolds, and that this differentiation is regulated by the ERK signaling pathway. Furthermore, the enhanced physical properties of collagen hydrogel scaffolds induced by the ECN treatment might play important roles in cell fate such as proliferation and differentiation. Therefore, within the limitations of the current study, the crosslinking of collagen with ECN seems to be beneficial for tissue engineering-based regenerative endodontic therapy.
